# Multilocus variable-number tandem-repeat analysis for genotyping of *Shigella sonnei* strains isolated from pediatric patients 

**Published:** 2015

**Authors:** Reza Ranjbar, Mojtaba Memariani

**Affiliations:** *Molecular Biology Research Center, Baqiyatallah University of Medical Sciences, Tehran, Iran*

**Keywords:** *Shigella sonnei*, multilocus variable-number tandem-repeat analysis, Iran

## Abstract

**Aim:**

The aims of this study were to characterize Iranian *Shigella sonnei* strains isolated from pediatric cases and evaluate the utility of multilocus variable-number tandem-repeat (VNTR) analysis (MLVA) for genotyping of local *S. sonnei* strains.

**Background:**

*S.* *sonnei* has become the dominant species in certain parts of Iran. Although PFGE is still a gold standard for genotyping and source tracking of food-borne pathogens, it is laborious, expensive, time-consuming, and often difficult to interpret. However, MLVA is a PCR-based method, which is rapid, relatively inexpensive and easy to perform.

**Patients and methods:**

A total of 47 *S. sonnei* isolates were obtained from sporadic cases of pediatric shigellosis in Tehran, Iran, during the years 2002-2003 (n=10) and 2008-2010 (n=37). The patients suffered from acute diarrhea and had evidence of more than three episodes of watery, loose, or bloody stools per day. A MLVA scheme based on 7 VNTR loci was established to assess the diversity of 47 *S. sonnei* isolates.

**Results:**

Based on the results, it was clear that the *S. sonnei* isolates were heterogeneous. Overall, 47 *S. sonnei* isolates were discriminated into 21 different genotypes. Analysis of the MLVA profiles using a minimum spanning tree (MST) algorithm showed the usefulness of the MLVA assay in discriminating *S. sonnei* isolates collected over different time periods. However, no correlation was found between the MLVA genotypes and age, gender or clinical symptoms of the patients.

**Conclusion:**

It is assumed that our *S. sonnei* isolates are derived from a limited number of clones that undergo minor genetic changes in the course of time. The present study has provided some valuable insights into the genetic relatedness of *S. sonnei* in Tehran, Iran.

## Introduction

 Shigellosis, also known as bacillary dysentery or Marlow’s Syndrome, continues to be a major cause of mortality and morbidity, especially in children with diarrhea in developing countries where there is overcrowding and poor sanitation (1). The genus *Shigella* comprises four species: *S. dysenteriae, S. flexneri*, *S. boydii*, and *S. sonnei*. With the exception of *S. sonnei*, each species may be further divided into several serotypes. Historically, *S. sonnei* is the predominant *Shigella* spp. in developed countries. However, recently, a change in trend has been reported from developing countries, where *S. flexneri* serotypes have been replaced by *S. sonnei* in areas undergoing economic development and improvements in hygiene (2-4).

Microbial genotyping is frequently applied for epidemiological investigations and provides useful information for establishing the genetic relatedness among pathogenic strains (5). In this regard, a number of genotyping methods have been developed for *S. sonnei*, including pulsed-field gel electrophoresis (PFGE) (6, 7, 8), multilocus enzyme electrophoresis (MLST) (9), multilocus variable-number tandem-repeat (VNTR) analysis (MLVA) (10, 11, 12, 13, 14), ribotyping (2, 15, 16, 17), plasmid profiling (5, 18), Repetitive Sequence-Based PCR (REP-PCR), and Enterobacterial Repetitive Intergenic Consensus sequence-based PCR (ERIC-PCR) (9, 19, 20). Among these methods, PFGE is still a gold standard for molecular subtyping and source tracking of food-borne pathogens. However, it is too discriminatory for clonal analysis of *S. sonnei* strains which have evolved over a longer time span (21). In addition, PFGE is laborious, expensive, time-consuming, often difficult to interpret, and requires rigorous standardization. Moreover, it needs experienced personnel in order to achieve reliable, consistent, and reproducible results. In contrast to PFGE, MLVA is a PCR-based genotyping method, which is rapid, relatively inexpensive and easy to perform. The method is based on the inherent variability of short sequences, which are organized as tandem repeats at multiple VNTR loci (12, 21).

Although *S. sonnei* is becoming an important etiologic agent of pediatric shigellosis in Iran (2, 3), there is limited information on the genetic background of the local strains. Therefore, we aimed to characterize *S. sonnei* isolates using a simple MLVA assay to evaluate the utility of this method for establishing phylogenetic relationships among *S. sonnei* strains in Iran, Tehran. 

## Patients and Methods

***Bacterial strains***

A total of 47 *S. sonnei* isolates were obtained from 950 patients (less than 12 years-of-age). These strains were isolated from sporadic cases of endemic shigellosis in Tehran, Iran, during the years 2002-2003 (n=10) and 2008-2010 (n=37). The children suffered from acute diarrhea and had evidence of more than three episodes of watery, loose, or bloody stools per day. Each strain was excluded from one patient. Shigellosis is confirmed through the culture of a stool specimen or rectal swab according to standard laboratory procedures. Briefly, the culture plates (MacConkey agar or XLD agar) were incubated overnight at 37°. Non-lactose fermenting colonies were selected and subjected to routine biochemical and serological tests. The serological test was carried out with commercial antisera (Mast Diagnostic, Merseyside, UK) by using slide agglutination method (2). The verified isolates were preserved at -70°C in Tripticase soy broth with 25% (v/v) glycerol for further analysis. The *S. sonnei* isolates were not repeatedly subcultured before this study to avoid any possible changes in the number of repeats within VNTR loci.

All ethical issues were considered. Life, health, dignity, integrity, right to self-determination, privacy, and confidentiality of personal information of research subjects were protected in this study.

***DNA preparation***

A pure culture of *S. sonnei* was plated on nutrient agar and incubated overnight at 37°C. A single colony was removed from the plate, suspended in 200 μl of sterile deionized water, and boiled for 15 min. After centrifugation at 8,000 g for 6 min, the supernatant was transferred into a new tube for subsequent PCR analysis.

**Table 1:** MLVA primers, Tandem Repeat (TR) sizes, and annealing temperatures for PCR reactions.

VNTR locus	Primer sequence (5' to 3')	Tandem Repeat (TR) size, bp	Ta °C
ms06	F: AAA CGG GAG AGC CGG TTA TT	39	55°C
	R: TGT TGG TAC AAC GGC TCC TG		
ms07(CVN001)	F: GTC AGT TCG CCC AGA CAC AG	39	55°C
	R: CGG TGT CAG CAA ATC CAG AG		
ms09	F: GTG CCA TCG GGC AAA ATT AG	179	55°C
	R: CCG ATA AGG GAG CAG GCT AGT		
ms11	F: GAA ACA GGC CCA GGC TAC AC	96	55°C
	R: CTG GCG CTG GTT ATG GGT AT		
ms21	F: GCT GAT GGC GAA GGA GAA GA	141	55°C
	R: GGG AGT ATG CGG TCA AAA GC		
ms23	F: GCT CCG CTG ATT GAC TCC TT	375	55°C
	R: CGG TTG CTC GAC CAC TAA CA		
ms32	F: GAG ATT GCC GAA GTG TTG C	101	55°C
	R: AAC TGG CGG CGT TTA TCA AG		

***MLVA assay***

The following seven VNTR loci were selected: ms06, ms07 (CVN001), ms09, ms11, ms21, ms23, and ms32. The primer sets for PCR amplification of these VNTR loci were previously reported by Gorge et al. (Table 1) (22). For each loci, PCR was performed in 25 μl volume containing 1X PCR buffer (50mmol/L KCL, 10 mmol/L Tris, pH9), 2.5 mmol/L MgCl_2_, 0.2 mmol/L of each primer with 0.5 U TaqDNA polymerase (CinnaGen Co., Iran), and 3 μl of DNA extract. Cycling conditions for PCR reactions were 93°C for 5 min, followed by 34 cycles of 93°C for 30 s, 55°C for 1 min, and 72°C for 1 min. The amplified products were run on agarose gels, stained with ethidium bromide, and visualized under UV transillumination.

***Data analysis***

**Figure 1:**
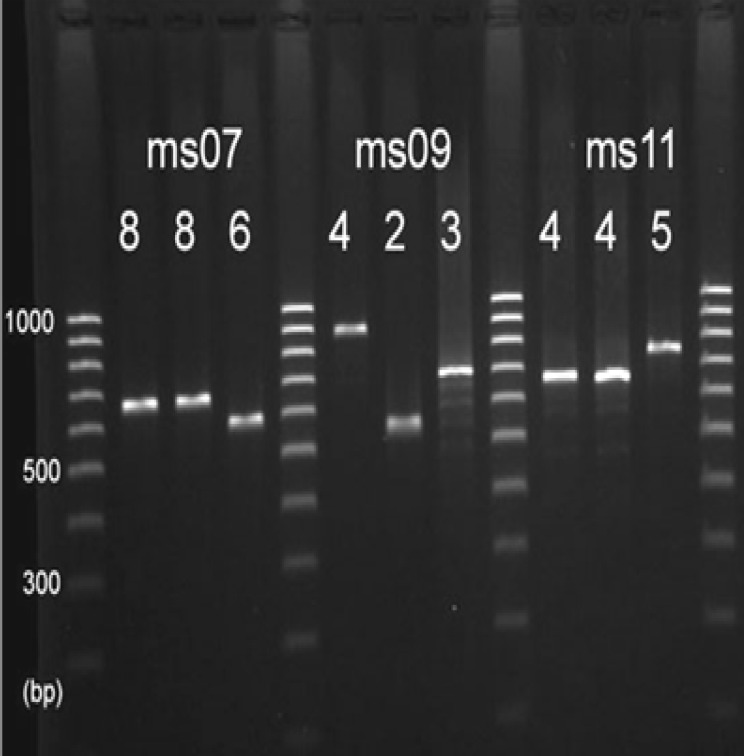
Polymorphism of 3 VNTR loci in different *S. sonnei* isolates. This image illustrates how the number of repeats can be directly deduced by manual reading. The numbers above the amplicons provide the repeat numbers of each VNTR locus.

The number of repeats can be easily deduced from the amplicon sizes by manual reading (Figure 1). Amplicon sizes were converted into numbers of repeats based on the formula: number of repeats (bp) = PCR product size (bp) − flanking regions (bp)/repeat size (bp). Repeat numbers were imported into Microsoft Excel. The minimum spanning tree (MST) was constructed with a categorical coefficient based on allelic profiles of the *S. sonnei* strains using trial version of Ridom MLVA compare software (Ridom® GmbH, Germany). MST is a convenient complementary tool to cluster multiple isolates and visualize the relative diversity within different lineages. A dendrogram of genetic relationships was also generated using the unweighted pair group method with arithmetic averages (UPGMA) method (23).

 Furthermore, Simpson's index of diversity (D) and 95 % confidence intervals (CI) for each VNTR locus were calculated using the V-DICE software (Health Protection Agency, London, UK; http:// www.hpa-bioinfotools.org.uk/cgi-bin/DICI/DICI.pl). 

## Results

Of all patients, 30 (52.6%) were females and 27 (47.4%) were males, with a female-to-male ratio of 1.1:1. The mean age of patients was 6.8±4.4SD. *S. sonnei* was isolated frequently from children under 5 years of age, who accounted for 59.6% (n=34) of all patients. About 40.5% (n=23) of the isolates recovered from persons aged 5-12 years. No correlation was found between the MLVA genotypes and age, gender or clinical symptoms of the patients. Fever (96.5%, n=55), nausea (79%, n=45), abdominal pain (77.2%, n=44), and convulsion (19.3%, n=11) were the most common clinical symptoms.

**Table 2:** Diversity indices and number of alleles have been shown for each VNTR locus.

VNTR locus	Simpson’s diversity of index	95% Confidence interval (CI)	No. of alleles	Detected alleles
ms06	0.520	0.479 - 0.560	3	2, 3, 4
ms07(CVN001)	0.569	0.487 - 0.651	6	2, 4, 5, 6, 7, 8
ms09	0.522	0.446 - 0.598	4	1, 2, 3, 4
ms11	0.194	0.049 - 0.339	3	4, 5, 6
ms21	0.534	0.475 - 0.593	3	4, 5, 6
ms23	0.506	0.444 - 0.568	2	1, 2
ms32	0.156	0.023 - 0.288	2	2, 4

**Figure 2:**
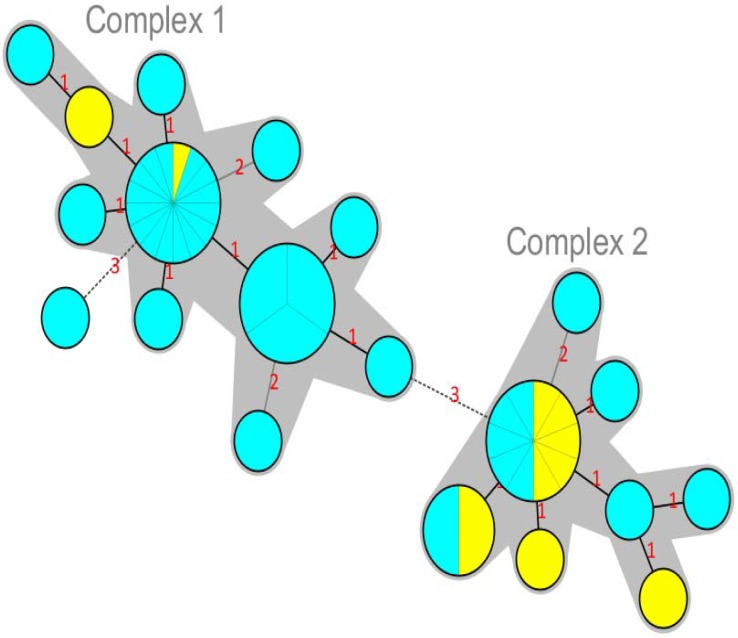
Minimum spanning tree (MST) for the 47 *S. sonnei* strains. Each circle represents one strain with a unique genotype (MLVA profile). The size of the circles indicates the number of isolates. The number of loci which differ between two MLVA types is indicated on the lines connecting the MLVA types. Clonal Complexes (CCs) were indicated by grey halos. The colours of the circles correspond to the date of isolation (yellow; 2002-2003, blue; 2008-2009).

**Figure 3:**
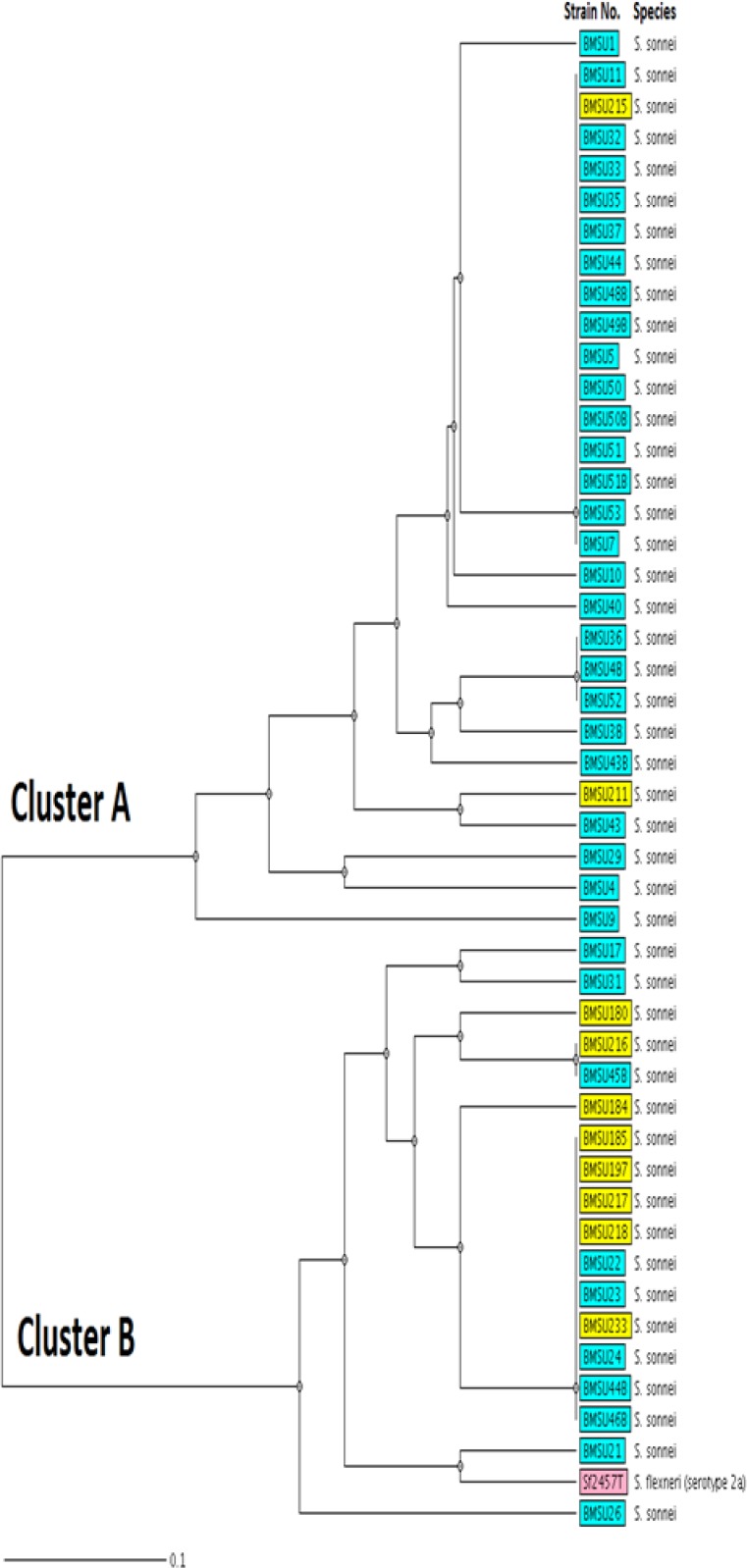
Clustering of the MLVA profiles by UPGMA with the categorical coefficient of similarity. The colours of each strain correspond to the date of isolation (yellow; 2002-2003, blue; 2008-2009). The dendrogram also includes the reference strain *S. flexneri* serotype 2a (Sf2457T).

MLVA based on seven VNTR loci was performed to characterize the *S. sonnei* strains. Overall, the 47 *S. sonnei* strains were discriminated into 21 different genotypes (MLVA types). As shown in Figure 2, almost all of the isolates were classified into two clonal complexes (CCs), while only one isolate was assigned as singleton. Clonal complexes/groups are defined as a group of allelic profiles in which every profile shares at least 5 loci in common with at least one other member of the group. Interestingly, MST analysis showed that most of the *S. sonnei* strains belonged to 2002-2003 period fell into CC2, whereas CC1 mainly comprised strains from the 2008-2009 period. Similarly, UPGMA analysis categorized the isolates into two main clusters (Figure 3). 

The number of alleles and diversity of each VNTR locus is presented in Table 2. The number of individual alleles ranged between 2 (ms32) and 6 (ms07). The diversity indices (D) of the VNTR loci differed considerably. Among the seven loci tested, locus ms32 showed the lowest diversity (D=0.156, [95% CI, 0.023 - 0.288]), while ms07 was the most diverse (D = 0.569, [95% CI, 0.478 -0.651]) (Table 2). 

## Discussion

*S. sonnei* is the predominant *Shigella* species isolated in industrialized countries, whereas *S. flexneri* predominates in developing countries. Overall, the situation in Iran agreed with this statement. 

In spite of that, in certain parts of Iran, such as Tehran, where the public hygiene and sanitation were good, a significant reduction in prevalence of *S. flexneri* compared with *S. sonnei* was observed (2, 3). In our study, *Shigella *strains were isolated frequently from children under 5 years of age, who accounted for 59.6% of all isolates. Previous studies showed that children are also at higher risk of getting the infection (2, 3, 18).

Based on the results, it was clear that our *S. sonnei* isolates were heterogeneous. Overall, 21 genotypes have been observed among 47 *S. sonnei* isolates. To our knowledge, there have been few studies regarding the genetic diversity of *S. sonnei* strains in Iran. Ranjbar et al. found only five ERIC-PCR patterns among 54 *S. sonnei* strains isolated from pediatric patients in Tehran (24). Another study showed the lack of ribotype diversity among *S. sonnei* strains recovered from hospitalized children in Tehran (2). PFGE has also been used for characterization of *S. sonnei*. Tajbakhsh et al. showed that seven distinct pulsotypes have been observed among 25 *S. sonnei* strains obtained from patients admitted to Milad Hospital, Tehran (3). In another study conducted in Shiraz, plasmid profile analysis identified 42 genotypes among 61 *S. sonnei* strains obtained from patients less than 14 years of age (18).

 Over the past few years, several MLVA schemes have been designed for *S. sonnei*. For instance, in a study carried out in Malaysia, 40 strains of *S. sonnei* strains isolated during the years 1997–2000, and 2007–2009 were classified into different clones based on seven VNTR loci. They also observed that *S. sonnei* isolates with no epidemiological linkage were clustered together which may be due to travel within the country and/or person-to-person spread of a particular strain over a long period of time with minor genetic changes (10). Moreover, they reported that there was no clear demarcation of the strains isolated from different years, which was almost in contrast with our results. In the current study, we showed the usefulness of our MLVA assay in discriminating *S. sonnei* strains collected over different time periods. In Japan, MLVA was also successfully applied to study the epidemiology of *S. sonnei* strains isolated from cases associated with foreign travel, and the correlations among molecular types, biotypes, resistance types and their geographical areas of origin. Interestingly, it was shown that *S. sonnei* isolates were classified into different clusters mainly on the basis of their countries of origin (13). Liang et al. developed and evaluated MLVA assay based on 26 VNTR loci for disease surveillance and outbreak investigations. They demonstrated that MLVA was able to discriminate PFGE-indistinguishable isolates (21). Chiou et al. also used the same VNTR loci for phylogenetic analysis of 916 *S. sonnei* isolates collected in Taiwan over 9 years (from 1996 to 2004). Three distinct clonal groups have been identified and the concordance level between MLVA and PFGE was remarkably high (12). Recently, Ingrid Filliol-Toutain et al. conducted a comprehensive study on the global epidemiology of *S. sonnei* using MLVA26, which was previously developed by Liang et al. They observed that 1672 *S. sonnei* isolates, which were obtained from 50 countries, including Iran, classified into four clonal groups (i.e. groups A to D). No common MLVA26 type was almost shared among isolates from different countries. Notably, all of the Iranian isolates fell into clonal group A (SS18.1 and SS18.2 MLVA types) which represented 1,382 isolates obtained since 1943 in 40 countries on 5 continents and the Paciﬁc region. Generally, these studies showed that MLVA has the potential to replace PFGE as a standard method of typing *S. sonnei* isolates. However, lack of standardization of the methodology and interpretive criteria is problematic and hinders comparison of data between laboratories.

Although our MLVA typing scheme for *S. sonnei* is less discriminatory than those of other studies (10, 11, 12, 13, 21), other schemes require a high precision of DNA length measurement, such as microcapillary electrophoresis and fluorescent markers. It is also worth mentioning that developing countries have limited accessibility to such equipments and their expenditures would be a major obstacle for many laboratories (22, 25). In our MLVA assay, we selected VNTR markers which can be easily analyzed by eye on agarose gels. Therefore, the assay can be carried out in a laboratory equipped with simple molecular biology equipment. On the other hand, VNTR loci have a wide range of evolutionary rates; thus, they can be exploited to investigate genetic relationships among isolates that have evolved over different timescales. Rapidly evolving VNTR loci with high variability are suitable for discerning closely related isolates or investigation of short-term epidemiology, such as disease outbreaks and disease surveillance, whereas slowly evolving VNTR loci with low variability are appropriate for establishing clearer clonal relationships among strains that have evolved over longer timescales (26).

In conclusion, it is assumed that our *S. sonnei* strains are derived from a limited number of clones that undergo minor genetic changes in course of time. However, a larger sample size from a variety of geographical regions will be needed to determine which loci provide sufficient resolution for disease surveillance and outbreak investigation. Furthermore, due to monomorphic nature of *S. sonnei *(27), a larger set of VNTR loci would be more favorable to obtain a clearer separation of clonal groups. Finally, this study has provided some valuable insights into the genetic relatedness of *S. sonnei* in Tehran, Iran.
